# The impact of exercise intervention on physical self-esteem of Chinese college students: a systematic review and meta-analysis

**DOI:** 10.3389/fpsyg.2026.1852169

**Published:** 2026-06-03

**Authors:** Mingyang Wang, Dongkai Li, Yiqi Song, Hao Kong, Fang Wu

**Affiliations:** 1 Department of Physical Education and Research, China University of Mining and Technology (Beijing), Beijing, China; 2 School of Physical Education, North China Institute of Science and Technology, Langfang, Hebei, China; 3 School of Athletic Training, Tianjin Sports Institute, Tianjin, China

**Keywords:** college students, exercise, intervention effect, meta-analysis, moderating factors, physical activity, physical self-esteem

## Abstract

**Objective:**

This study systematically synthesized relevant empirical research through meta-analysis to quantify the intervention effects of different exercise modalities on college students’ physical self-esteem, identify key moderating variables driving between-study heterogeneity, and provide an evidence-based basis for colleges and universities to develop precise exercise intervention programs.

**Methods:**

In accordance with the PRISMA 2020 guidelines, we systematically searched six electronic databases (CNKI, Wanfang Data, Embase, PubMed, Web of Science, and the Cochrane Library) from their inception to September 7, 2025 to identify eligible randomized controlled trials. The Cochrane Risk of Bias 2 (Rob 2) tool was adopted to evaluate the risk of bias of the included studies, the GRADE system was used to rate the quality of evidence for all core physical self-esteem-related outcomes, and effect size pooling, predefined subgroup analysis, and meta-regression were performed to explore sources of between-study heterogeneity. The study protocol was retrospectively registered with PROSPERO (Registration No. CRD420261356511).

**Results:**

A total of 12 studies involving 2,128 participants were included. Meta-analysis using a random-effects model showed that exercise intervention had a significant large positive effect on the overall level of college students’ physical self-esteem (SMD = 0.95, 95% CI [0.70, 1.20], *p* < 0.001), with substantial between-study heterogeneity (*I*^2^ = 79, 95% prediction interval [0.12, 1.78]). Exercise interventions also exerted significant moderate positive impacts on all core dimensions of physical self-esteem: physical self-worth (SMD = 0.55, 95% CI [0.39, 0.71], *p* < 0.001), sport competence (SMD = 0.68, 95% CI [0.54, 0.83], *p* < 0.001), physical condition (SMD = 0.67, 95% CI [0.55, 0.78], *p* < 0.001), physical attractiveness (SMD = 0.68, 95% CI [0.50, 0.85], *p* < 0.001), and physical fitness (SMD = 0.64, 95% CI [0.47, 0.81], *p* < 0.001). Predefined subgroup analyses identified preliminary indications that intervention effects may be greater in males, those receiving combined aerobic–resistance training, and healthy individuals. Sensitivity analysis confirmed the robustness of the core qualitative conclusion, but publication bias was detected for most outcomes, and the certainty of evidence ranged from low to moderate according to the GRADE system.

**Conclusion:**

Current available evidence suggests that exercise interventions may improve overall physical self-esteem and all its dimensions in Chinese college students. The subgroup analyses provide preliminary indications that greater benefits may be observed in males, those receiving combined aerobic and resistance training, and healthy individuals. However, these subgroup findings are hypothesis-generating rather than definitive, and the pooled effect estimate should be interpreted with caution due to substantial between-study heterogeneity and low-to-moderate evidence quality. Future research should expand sample sizes, standardize exercise intervention parameters, and conduct head-to-head trials to verify these preliminary subgroup effects.

## 1 Introduction

As a population undergoing the transition from late adolescence to early adulthood, college students face critical developmental challenges related to self-identity formation and mental health maintenance. In recent years, the college period has increasingly become a high-stress stage for many students, owing to the growing number of graduates, rapid social change, and intensified employment competition (Auerbach et al., 2016). This mounting stress has been accompanied by a rising prevalence of mental health problems among college students (Yuan et al., 2025). Accordingly, addressing mental health concerns and promoting the integrated development of physical and psychological well-being have become important responsibilities for Chinese universities, both in fulfilling their educational mission and in improving the quality of talent cultivation.

In psychological research, self-esteem is regarded as a key construct with strong predictive value for changes in emotional states and personality development, and it is also considered essential for mental health (Sowislo and Orth, 2013). As an important component of global self-esteem, physical self-esteem—alongside academic and interpersonal self-esteem—plays a central role in the overall structure of self-evaluation. Importantly, physical self-esteem does not reflect an objective assessment of bodily characteristics; rather, it refers to individuals’ subjective evaluations and emotional experiences regarding their physical attributes, functional capacities, and outward performance, including perceptions of body image, motor competence, and related domains (Gualdi-Russo et al., 2022; Pop et al., 2022; Aparicio-Martinez et al., 2019). This construct was first conceptualized by Fox through the Physical Self-Perception Profile (PSPP) (Fox and Corbin, 1989), which emphasized the affective core of physical self-concept. Xia and Jiaxin (2001) subsequently validated the construct in Chinese college students and proposed five dimensions of physical self-esteem: physical self-worth, sport competence, physical condition, physical attractiveness, and physical fitness. These dimensions primarily capture subjective perceptions and are not directly equivalent to objective physical indicators (Gualdi-Russo et al., 2022; Pop et al., 2022; Kokoszka et al., 2022). Previous studies have shown that higher physical self-esteem may enhance overall self-esteem (Patel et al., 2025) and help buffer a range of mental health problems, including emotional dysregulation (Abdoli et al., 2025), eating disorders (Şenay and Yücel, 2023), aggressive behaviors (Xiaoning et al., 2023), and interpersonal difficulties (Chao and Guoli, 2020). Identifying effective strategies to enhance physical self-esteem may therefore have important implications for promoting mental health in college populations.

Current research has identified several approaches for improving physical self-esteem, including cognitive interventions, group counseling, and exercise-based interventions. Among these, exercise interventions have received substantial empirical support (Earl, 2023; Lopes et al., 2022). However, most existing studies are individual empirical investigations, and robust quantitative synthesis remains limited. In particular, the relative effectiveness of different exercise modalities remains unclear, and there is insufficient evidence regarding whether intervention effects vary across subgroups of college students. Moreover, potentially important moderators, such as gender and health status, have not been systematically quantified, which limits the precise application of exercise interventions in university settings.

Against this background, the present study conducted a meta-analysis to systematically synthesize empirical evidence on the relationship between exercise interventions and physical self-esteem among college students. Specifically, we pooled the overall effects of exercise interventions on physical self-esteem and examined whether intervention effects varied according to key moderator variables, including exercise modality, intervention duration, frequency, and health status. By clarifying the overall effectiveness of exercise interventions and exploring potential sources of between-study heterogeneity, this study aims to provide both a theoretical basis and quantitative evidence to inform the development of targeted exercise intervention programs in university settings.

## 2 Materials and methods

This systematic review and meta-analysis were conducted and reported in accordance with the Preferred Reporting Items for Systematic Reviews and Meta-Analyses (PRISMA) 2020 statement. The study protocol was retrospectively registered in the International Prospective Register of Systematic Reviews (PROSPERO) under registration number CRD420261356511. The full protocol is publicly available at: https://www.crd.york.ac.uk/PROSPERO/view/CRD420261356511. All major methodological procedures reported in the present review were conducted in accordance with the registered protocol.

### 2.1 Literature search

Two reviewers independently searched six electronic databases: PubMed, Embase, Wanfang Data, CNKI, Web of Science, and the Cochrane Library. The search covered each database from inception to 7 September 2025. For the Chinese-language databases, the search terms were as follows: (“Exercise” OR “Physical Activity” OR “Physical Education”) AND (“Self-Esteem” OR “Physical Self-Esteem” OR “Body Satisfaction” OR “Self-Body Evaluation”) AND (“College Students” OR “Colleges and Universities” OR “Youth”). A comparable strategy was applied to the English-language databases using the terms (“Exercise” OR “Physical Activity” OR “Physical Education”) AND (“Self-Esteem” OR “Physical Self-Esteem” OR “Body Satisfaction” OR “Self-Body Evaluation”) AND (“College Students” OR “Colleges and Universities” OR “Young Adults”). To minimize the risk of missing eligible studies, we also manually screened the reference lists of the included articles, as well as relevant cited and related records. The detailed search strategies for each database are presented in Table 1.

**Table 1:** Literature search strategy.

Database	Search strategy	Number
PubMed	Title/Abstract = (Exercise OR Physical Activity OR Physical Education) AND (Self-Esteem OR Physical Self-Esteem OR Body Satisfaction OR Self-Body Evaluation) AND (College Students OR Colleges and Universities OR Young Adults)	63
Embase	Broad search = (Exercise OR Physical Activity OR Physical Education) AND Broad search = (Self-Esteem OR Physical Self-Esteem OR Body Satisfaction OR Self-Body Evaluation) AND Broad search = (College Students OR Colleges and Universities OR Young Adults)	183
WanFang	Topic = (Exercise OR Physical Activity OR Physical Education) AND (Self-Esteem OR Physical Self-Esteem OR Body Satisfaction OR Self-Body Evaluation) AND (College Students OR Colleges and Universities OR Young Adults)	1904
CNKI	Topic = (Exercise OR Physical Activity OR Physical Education) AND (Self-Esteem OR Physical Self-Esteem OR Body Satisfaction OR Self-Body Evaluation) AND (College Students OR Colleges and Universities OR Young Adults)	1,139
WOS	TS = (Exercise OR Physical Activity OR Physical Education) AND TS = (Self-Esteem OR Physical Self-Esteem OR Body Satisfaction OR Self-Body Evaluation) AND TS = (College Students OR Colleges and Universities OR Young Adults)	239
Cochrane Library	(Title/Abstract/Keyword = Exercise OR Physical Activity OR Physical Education) AND (Title/Abstract/Keyword = Self-Esteem OR Physical Self-Esteem OR Body Satisfaction OR Self-Body Evaluation) AND (Title/Abstract/Keyword = College Students OR Colleges and Universities OR Young Adults)	378

### 2.2 Inclusion criteria

The inclusion and exclusion criteria were defined according to the PICOS framework. The inclusion criteria were as follows: (1) Population (P): Junior college, undergraduate and postgraduate students enrolled in colleges and universities. (2) Intervention (I): Any form of exercise intervention, with participants in the experimental group assigned to an exercise program. (3) Comparison (C): A non-intervention control condition, defined as no targeted exercise intervention, medication, group counseling, or other structured intervention. (4) Outcome (O): Physical self-esteem assessed using the Physical Self-Perception Profile (PSPP), originally developed by Fox and later revised for the Chinese population by Xia and Jiaxin (2001). The PSPP evaluates individuals’ subjective perceptions of the physical self and includes one global scale and four subdimensions. The global scale, Physical Self-Worth (PSW), assesses overall pride, satisfaction, enjoyment, and confidence in relation to one’s body. The four subdimensions are Sport Competence (SC), which assesses perceived sport ability, acquisition of sport-related skills, and confidence in sport settings; Physical Condition (PC), which evaluates perceived physical condition and confidence in physical performance; Physical Attractiveness (PA), which measures perceived attractiveness and confidence in physical appearance; and Physical Fitness (PF), which assesses self-perceived fitness attributes such as speed, endurance, strength, and flexibility. Each subdimension contains six items rated on a 4-point Likert scale, ranging from 1 (“not at all”) to 4 (“completely”), yielding subdimension scores from 6 to 24, with higher scores indicating more positive physical self-perceptions in that domain. (5) Study design (S): Randomized controlled trials and randomized crossover trials.

### 2.3 Literature screening

Two reviewers independently screened the retrieved records according to the predefined search strategy and eligibility criteria. Duplicate records were removed using EndNote. In the initial screening stage, titles, keywords, and abstracts were reviewed to exclude studies that were clearly irrelevant to the research question. The remaining articles were then assessed in full text against the predefined inclusion and exclusion criteria. Studies that did not meet the eligibility criteria were excluded, and the reasons for exclusion were documented. The screening decisions made by the two reviewers were subsequently cross-checked to determine the final set of included studies. Any disagreements were resolved through discussion, and when necessary, consultation with a third reviewer.

### 2.4 Data extraction

Two reviewers independently extracted data from all included studies using a pre-piloted standardized form. The extracted data included: (1) basic study characteristics, including first author, publication year, and study design; (2) participant characteristics, including sample size, gender distribution, and baseline health status; (3) exercise intervention characteristics, including intervention modality, exercise intensity, session duration, weekly frequency, total intervention duration, and specific exercise protocol; and (4) outcome data, including the post-intervention mean, standard deviation, and sample size for physical self-esteem outcomes in both the experimental and control groups.

For studies that included multiple independent intervention groups sharing a common control group, each intervention–control comparison was treated as an independent effect size in accordance with the recommendations of the Cochrane Handbook for Systematic Reviews of Interventions. This approach allowed all relevant intervention data to be incorporated while avoiding inappropriate duplication of participants in the shared control group. Any discrepancies in data extraction were resolved through discussion and, when necessary, consultation with a third senior reviewer.

### 2.5 Risk of Bias and methodological quality assessment

The risk of bias of the included randomized controlled trials was independently assessed by two reviewers using the Cochrane Risk of Bias 2 (Rob 2) tool. Five domains were evaluated: the randomization process, deviations from intended interventions, missing outcome data, measurement of the outcome, and selection of the reported result. Before the formal assessment, the reviewers received training to ensure consistency and methodological rigor. Each reviewer independently completed the assessment forms and documented the rationale for their judgments. Any disagreements were resolved through discussion, and when necessary, a third reviewer was consulted for arbitration.

### 2.6 GRADE evidence quality assessment

The certainty of evidence for six key outcomes related to the effects of exercise interventions on physical self-esteem in college students was evaluated using the Grading of Recommendations Assessment, Development and Evaluation (GRADE) approach. These outcomes included the total physical self-esteem score and five subdimensions. Evidence was assessed for possible downgrading across five domains: risk of bias, inconsistency, indirectness, imprecision, and publication bias. The certainty of evidence was classified into four levels: high, moderate, low, and very low (Guyatt et al., 2011). All assessments were conducted independently by two reviewers, and any disagreements were resolved through discussion or, when necessary, arbitration by a third senior reviewer.

### 2.7 Statistical analysis

Statistical analyses were conducted using R (version 4.3.3) and Stata 15.0. To estimate the effects of exercise interventions relative to control conditions on overall physical self-esteem and its subdimensions, effect sizes were pooled using the generic inverse-variance method with the DerSimonian–Laird random-effects model (DerSimonian and Laird, 1986). This estimator was selected for three reasons: it is widely used in exercise and sport psychology meta-analyses, it accounts for expected between-study heterogeneity, and it provides stable, interpretable, and reproducible pooled estimates. Effect sizes were expressed as standardized mean differences (SMDs) and calculated as Hedges’ g to correct for small-sample bias. Effect sizes were interpreted according to the following predefined thresholds: negligible (<0.2), small (0.2 to < 0.5), moderate (0.5 to < 0.8), and large (≥0.8) (Hedges et al., 2010). Statistical significance was set at a two-tailed *α* level of 0.05.

Between-study heterogeneity was assessed using Cochran’s Q test and the *I^2^* statistic. Heterogeneity was interpreted as low (< 25%), moderate (25% to < 50%), high (50% to < 75%), or very high (≥ 75%). For secondary outcomes with no evidence of substantial heterogeneity, fixed-effect models were applied; otherwise, the prespecified DerSimonian–Laird random-effects model was retained for outcomes with significant heterogeneity (Q test *p* ≤ 0.10 or *I^2^* ≥ 50%). Pooled effect estimates were reported with 95% confidence intervals (CIs). In addition, to evaluate the potential influence of between-study heterogeneity on the generalizability of the findings, 95% prediction intervals (PIs) were calculated for the primary outcome using the t distribution to estimate the likely range of true effects across different study settings and intervention contexts (Nagashima et al., 2019). Statistical outliers were defined as studies whose 95% CIs did not overlap with the 95% CI of the overall pooled effect.

To explore prespecified sources of heterogeneity and examine the influence of study-level characteristics on intervention effects, meta-regression and subgroup analyses were performed in Stata 15.0. Univariate meta-regression with the mixed command was first conducted to identify potential sources of between-study heterogeneity; for variables showing directional trends, subgroup analyses via the metan module were performed, with between-group differences tested by the Q test for interaction.

Sensitivity analyses were conducted using a leave-one-out approach, in which each study was sequentially removed and the pooled effect size and corresponding 95% CI were recalculated. The robustness of the main findings was evaluated by examining changes in the pooled estimate after exclusion of each individual study.

Publication bias was assessed using funnel plots and Egger’s linear regression test. Funnel plots were first generated for visual inspection of asymmetry, followed by Egger’s test implemented in Stata, with *p* > 0.05 indicating no statistically significant evidence of publication bias.

Pre–post change scores were calculated using standard formulas recommended in the Cochrane Handbook for Systematic Reviews of Interventions. When pre–post correlation coefficients were not reported in the original studies, an assumed correlation coefficient of r = 0.5 was applied as the default value.

$$M_{\text{change}}=M_{\text{Post}-\text{test value}}-M_{\mathrm{Pre}-\text{test value}}$$

$${\mathrm{SD}}_{\text{Change}}=\sqrt{\begin{array}{l} {{\mathrm{SD}}^{2}}_{\mathrm{Pre}-\text{test value}}+{{\mathrm{SD}}^{2}}_{\text{Post}-\text{test value}}-\\ \left(2\times r\times {\mathrm{SD}}_{\mathrm{Pre}-\text{test value}}\times {\mathrm{SD}}_{\text{Post}-\text{test value}}\right) \end{array}}$$

$$\mathrm{ES}=\;\sqrt{\begin{array}{l} \frac{\left(\begin{array}{l} M_{\text{Changes in the intervention group}}-\\ M_{\text{Changes in the control group}} \end{array}\right)}{{\mathrm{SD}}_{\text{pooled}}}\\ \times (1-\frac{3}{4(n_{\text{Intervention}}+n_{\text{Control}}-2)-1}) \end{array}}$$

$$\mathrm{SD}=\mathrm{SE}\times \sqrt{N}.$$

Note: M = mean; SD = standard deviation; ES = effect size; n = sample size.

## 3 Results

### 3.1 Literature search results

**Figure 1:**
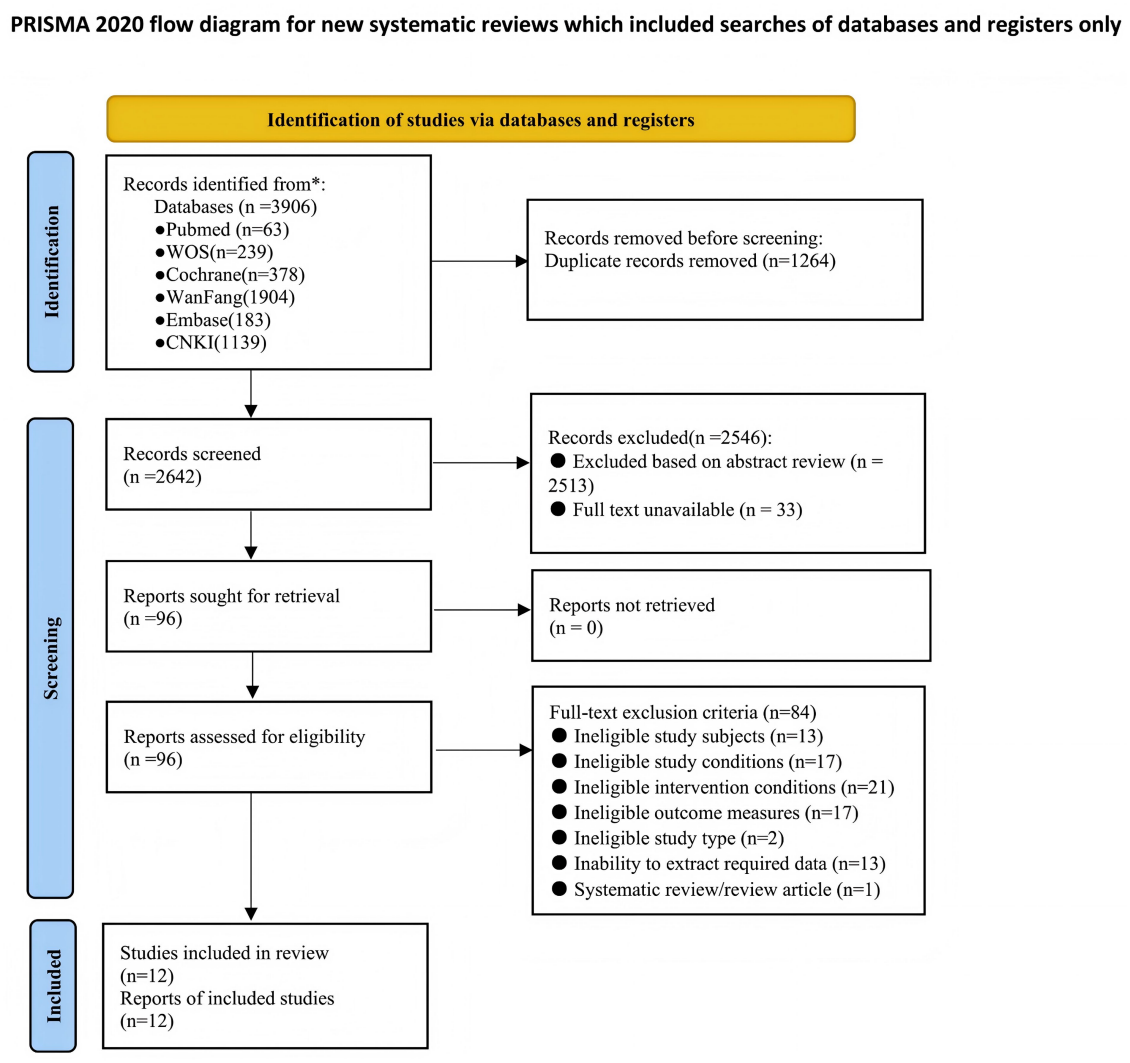
Literature screening flowchart.

The initial search of the six databases yielded a total of 3,906 records, including CNKI (*n* = 1,139), Wanfang Data (*n* = 1,904), Embase (*n* = 183), Web of Science (*n* = 239), PubMed (*n* = 63), and the Cochrane Library (*n* = 378). All records were imported into Note Express for reference management. After automatic and manual deduplication, 1,264 duplicate records were removed. The remaining records were screened by title, abstract, and keywords, resulting in the exclusion of 2,546 articles. Ninety six full-text articles were then assessed against the predefined eligibility criteria, and 84 were excluded. Finally, 12 studies were ultimately included in the meta-analysis (He Ying, 2003; Chunyī, 2009; Jinfeng, 2010; Xiaobo, 2013; Yajun, 2016; Yunyan et al., 2019; Jianbo and Aihua, 2019; Hengzheng et al., 2020; Yuxin, 2021; Xiao-di and Yue-feng, 2022; Jun, 2024; Huan-wei, 2022). The literature screening process is presented in Figure 1.

### 3.2 Evaluation of the quality of the included literature

**Figure 2:**
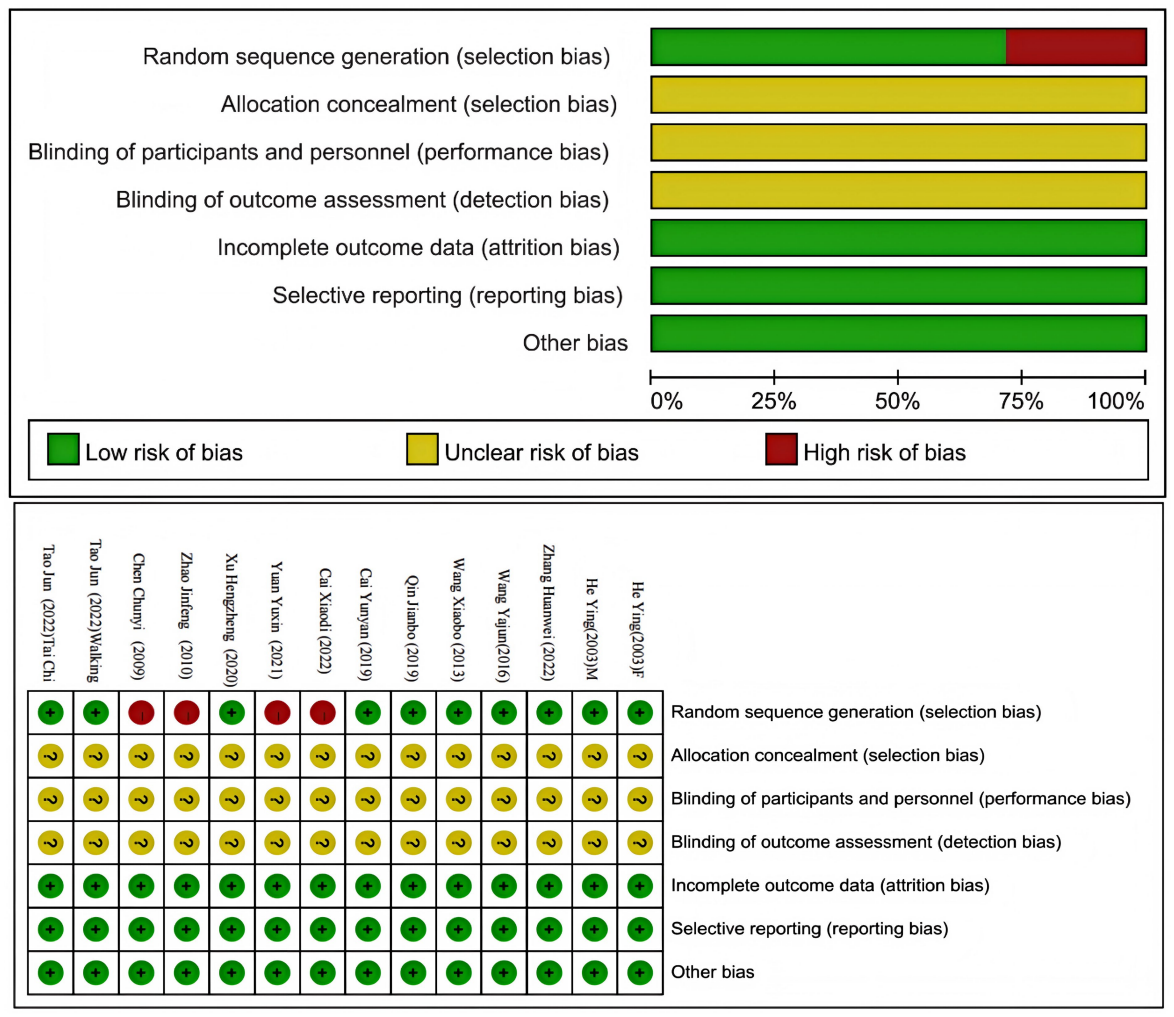
Literature quality assessment.

The methodological quality of the included studies was assessed using the Cochrane Risk of Bias 2 (RoB 2) tool in Review Manager 5.3 (Figure 2). All included studies were judged to be at low risk of bias for incomplete outcome data, selective reporting, and other sources of bias. Regarding random sequence generation, 67% of studies were rated as low risk of bias, whereas the remaining studies were judged to have potential bias because of insufficient reporting of randomization procedures. In addition, all studies were rated as having unclear risk of bias for allocation concealment, blinding of participants and personnel, and blinding of outcome assessment, as none explicitly reported standardized blinding procedures. Overall, the included studies were considered to be of acceptable methodological quality. Most core domains were rated as low risk, although incomplete reporting of allocation concealment and blinding remained a common concern, which should be taken into account when interpreting the pooled findings.

### 3.3 Basic characteristics of included studies

A total of 12 studies involving 2,128 participants were included in this review. Gender distribution was not reported in 3 studies, comprising 196 participants (9.2%). Among the remaining studies, 899 participants were male (42.6%) and 1,013 were female (48.0%). Four studies, involving 276 participants (13.0%), included individuals with specific health conditions, such as obesity, depression, or physical frailty. Regarding outcome reporting, 1 study (*n* = 120) reported only the total PSPP score as the outcome measure, 1 study (*n* = 479) provided data exclusively for the five PSPP subdimensions, and the remaining 10 studies (*n* = 1,509) presented complete data for both the total score and all subdimensions.

The interventions comprised aerobic exercise, resistance training, and combined aerobic–resistance training. Nine studies used moderate-intensity exercise (50–80% of maximum heart rate). Across studies, intervention duration ranged from 8 to 16 weeks, with sessions lasting 40–90 min and occurring 2–3 times per week. Detailed characteristics of the included studies are presented in Table 2.

**Table 2:** Basic characteristics of the included literature.

Author	Year	Participant characteristics				Exercise intervention program						
		Health status	Sample (*n*)	Male (*n*)	Female (*n*)	Exercise modality	Intensity	Duration (min)	Frequency (times/week)	Cycle (Week)	Specific exercise plan	Outcome
He Ying (2003)	2003	Depression	120	60	60	Aerobic exercise	NR	60	3	16	Jogging, climbing stairs	①
Chunyī (2009)	2009	Health	480	480	0	Aerobic exercise	NR	NR	NR	16	Basketball Skills and Tactics Training, Specialized Physical Conditioning, Simulated Games, Basketball Games	① + ②
Jinfeng (2010)	2010	obesity	60	0	60	Aerobic exercise	Moderate intensity	60	3	10	Aerobic Exercise Routine Practice, Rhythmic Full-Body Workout	① + ②
Xiaobo (2013)	2013	Health	30	30	0	Resistance training	NR	90	2	15	Half Squat, Seated Rowing, Flyes, Bench Press, Pull-ups, Standing Calf Raises, Dumbbell Curls, Weighted Sit-ups	① + ②
Yajun (2016)	2016	Health	195	88	107	Combining aerobic and resistance training	Moderate intensity	50	3	12	Jogging + Resistance Training (Push-ups, Sit-ups, Pull-ups)	① + ②
Yunyan et al. (2019)	2019	Health	479	0	479	Aerobic exercise	Moderate intensity	50 ~ 60	3	16	Aerobics, ballroom dancing, running	②
Jianbo and Aihua (2019)	2019	Health	280	139	141	Combining aerobic and resistance training	Moderate intensity	45 ~ 50	3	16	Aerobic exercise (jogging, aerobics) + Strength training (sit-ups, push-ups, pull-ups)	① + ②
Hengzheng et al. (2020)	2020	Weak constitution	56	NR	NR	Aerobic exercise	Moderate intensity	40 ~ 60	3	8	Eight Brocades	① + ②
Yuxin (2021)	2021	Health	60	0	60	Aerobic exercise	Moderate intensity	90	2	16	Cha-cha, Rumba (Basic Steps + Combinations, Solo / Partner Practice)	① + ②
Xiao-di and Yue-feng (2022)	2022	Health	208	102	106	Combining aerobic and resistance training	Moderate intensity	50	3	12	Running, soccer, basketball, badminton, aerobics, jump rope, sit-ups, pull-ups, push-ups	① + ②
Jun (2024)	2024	Weak constitution	60	NR	NR	Aerobic exercise	Moderate intensity	40 ~ 45	3	16	24-Form Simplified Tai Chi; Brisk Walking on the Track	① + ②
Huan-wei (2022)	2022	Health	100	NR	NR	Aerobic exercise	Moderate intensity	90	2	16	Aerobic Exercise Routine Practice, Rhythmic Full-Body Joint Movement	① + ②

NR, not reported; ① = Only the total Physical Self-Perception Profile (PSPP) score was reported; ② = Only the five subdimensions of the PSPP were reported; ① + ② = Both the total PSPP score and all five subdimensions were reported.

### 3.4 Meta-analysis results

The total score of the Physical Self-Esteem Scale was designated as the primary outcome. A total of 13 independent effect sizes were extracted from 11 of the 12 included studies. The additional effect size came from the study by Jun (2024), which included two independent intervention groups (24-form Tai Chi and brisk walking) that shared a single non-exercise control group; accordingly, each intervention–control comparison was treated as an independent effect size.

For the primary outcome, heterogeneity testing showed Tau^2^ = 0.15, χ^2^ = 56.75 (df = 12, *p* < 0.001), and *I^2^* = 79%, indicating very high between-study heterogeneity according to the predefined criteria. Using the prespecified random-effects model, exercise interventions were associated with significantly higher overall physical self-esteem than the non-exercise control condition (SMD = 0.95, 95% CI [0.70, 1.20], p < 0.001), representing a large effect size. The 95% prediction interval for the primary outcome ranged from 0.12 to 1.78, suggesting that the true intervention effect in a new study setting may vary from a small positive effect to a very large positive effect, depending on the population and intervention context.

The five subdimensions of the PSPP were treated as secondary outcomes, with 12 independent effect sizes extracted from 11 of the 12 included studies. Heterogeneity testing showed that *I^2^* values for the subdimensions ranged from 27 to 64% (*p* < 0.05 for all dimensions except physical condition), indicating moderate to high between-study heterogeneity. Accordingly, random-effects models were applied to all subdimension meta-analyses. The pooled results showed that exercise interventions produced significant improvements across all subdimensions, with effect sizes in the moderate range: physical self-worth (SMD = 0.55, 95% CI [0.39, 0.71], Z = 6.64, *p* < 0.001), sport competence (SMD = 0.68, 95% CI [0.54, 0.83], Z = 9.24, *p* < 0.001), physical condition (SMD = 0.67, 95% CI [0.55, 0.78], Z = 11.34, *p* < 0.001), physical attractiveness (SMD = 0.68, 95% CI[0.50, 0.85], Z = 7.45, *p* < 0.001), and physical fitness (SMD = 0.64, 95% CI [0.47, 0.81], Z = 7.34, *p* < 0.001).

**Figure 3:**
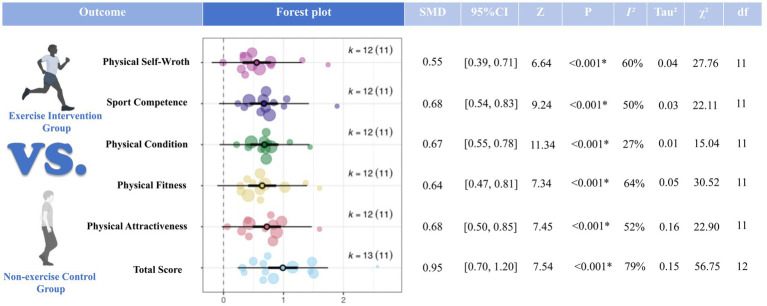
Meta-analysis results of exercise on PSPP total score and dimensions. Note: *k* = number of independent effect sizes; numbers in parentheses indicate the number of original included studies; *Z* denotes the *Z*-test statistic; *P* represents the *p* value for the statistical significance of the intervention effect; *I*^2^ indicates the percentage of between-study heterogeneity; Tau^2^ signifies the estimated between-study variance; χ^2^ denotes the Chi-square statistic for heterogeneity testing; *df* denotes its degrees of freedom; all *p* values < 0.001 are uniformly reported as *p* < 0.001.

Taken together, these findings indicate that exercise interventions were associated with consistent and statistically significant improvements in both overall physical self-esteem and its subdimensions (Figure 3). However, the very high between-study heterogeneity observed for the primary outcome warrants cautious interpretation of the pooled effect size. We therefore conducted prespecified subgroup analyses and meta-regression to further explore potential sources of heterogeneity.

### 3.5 Meta-regression results

Given the very high between-study heterogeneity observed for the total physical self-esteem score (*I^2^* = 79%), we conducted prespecified univariate meta-regression analyses to identify potential moderators and quantify the proportion of heterogeneity explained by each study-level variable. To preserve statistical power and minimize the risk of multiple testing across subdimensions, all meta-regression analyses were restricted to the total score. The null model indicated a baseline between-study variance of τ^2^ = 0.1918, with a residual *I^2^* of 78.86%.

As shown in Table 3, gender explained the largest proportion of between-study variance (adjusted R^2^ = 45.16%) and demonstrated a borderline significant moderating effect (*p* = 0.064). Exercise modality was identified as a statistically significant moderator (*p* = 0.046), accounting for 35.23% of the observed heterogeneity. Health status explained 21.39% of the variance and showed a directional pattern consistent with the subgroup analyses, although this effect did not reach statistical significance (*p* = 0.131). In contrast, weekly exercise frequency and single-session duration were not significant moderators (both *p* > 0.05) and explained little to no between-study variance.

**Table 3:** Univariate meta-regression analyses of moderators for the total effect of exercise on physical self-esteem.

Moderator	Coefficient	Standard error	95% CI	*p*	Adjusted R^2^ (%)
Gender	−0.23	0.11	[−0.47, 0.01]	0.064	45.16
Exercise modality	0.53	0.23	[0.01, 1.04]	0.046*	35.23
Health status	0.46	0.28	[−0.16, 1.09]	0.131	21.39
Single session duration	−0.29	0.34	[−1.05, 0.46]	0.406	8.91
Exercise frequency	−0.09	0.18	[−0.47, 0.30]	0.638	−16.27

Meta-regression analyses used REML with Knapp-Hartung correction; *p < 0.05; Adjusted *R*^2^ = proportion of between-study variance explained.

Overall, the three prespecified moderators—gender, exercise modality, and health status—accounted for a substantial proportion of the observed between-study heterogeneity. Nevertheless, some residual heterogeneity remained unexplained by the available study-level characteristics.

### 3.6 Subgroup analysis results

To explore potential sources of heterogeneity, subgroup analyses were conducted according to gender, intervention modality, health status, single-session duration, and weekly exercise frequency. Given the small overall number of included studies and the very limited sample sizes within most subgroups, all subgroup findings should be interpreted as preliminary, exploratory, and hypothesis-generating rather than definitive. The results suggested that gender, exercise modality, and health status were the main potential contributors to between-study heterogeneity. These factors were therefore examined in greater detail in the subsequent analyses (Tables 4–6).

**Table 4:** Effect of gender on total PSPP score and dimensions.

Dimension	Category	*n*	SMD	95% CI	*I^2^* (%)	Pi	*p*
Total score	Male Female	3 3	1.15 0.55	[0.98, 1.33] [0.25, 0.85]	81.6% 0.00%	0.004 0.548	0.001**
Physical self-worth	Male Female	2 3	0.65 0.41	[0.47, 0.83] [0.25, 0.58]	86.0% 14.0%	0.008 0.312	0.053
Sport competence	Male Female	2 3	0.82 0.48	[0.64, 1.00] [0.31, 0.64]	84.9% 60.7%	0.010 0.078	0.006**
Physical condition	Male Female	2 3	0.83 0.48	[0.65, 1.01] [0.31, 0.64]	61.8% 0.00%	0.106 0.807	0.005**
Physical attractiveness	Male Female	2 3	0.92 0.46	[0.74, 1.10] [0.30, 0.62]	67.0% 0.00%	0.082 0.422	0.000**
Physical fitness	Male Female	2 3	0.73 0.40	[0.55, 0.91] [0.24, 0.56]	79.4% 0.00%	0.028 0.896	0.007**

*n*, Number of included studies; *I*^2^, Percentage of between-study heterogeneity; Pi, *p* value for within-subgroup heterogeneity test; *p*, *p* value for between-subgroup difference test. *p < 0.05, **p < 0.01.

**Table 5:** Effect of intervention modality on PSPP total score and dimensions.

Dimension	Category	*n*	SMD	95% CI	*I^2^* (%)	Pi	*p*
Total score	Aerobic Aerobic combined with resistance training	9 3	0.88 1.20	[0.75, 1.01] [1.03, 1.36]	65.6% 87.1%	0.003 0.001	0.000**
Physical self-worth	Aerobic Aerobic combined with resistance training	8 3	0.48 0.59	[0.28, 0.67] [0.38, 0.80]	53.3% 46.6%	0.036 0.154	0.034*
Sport competence	Aerobic Aerobic combined with resistance training	8 3	0.64 0.69	[0.45, 0.83] [0.54, 0.84]	50.1% 0.00%	0.051 0.927	0.040*
Physical condition	Aerobic Aerobic combined with resistance training	8 3	0.63 0.70	[0.47, 0.80] [0.55, 0.85]	36.7% 0.00%	0.136 0.982	0.031*
Physical attractiveness	Aerobic Aerobic combined with resistance training	8 3	0.53 0.89	[0.31, 0.75] [0.73, 1.04]	63.1% 0.00%	0.008 0.676	0.004**
Physical fitness	Aerobic Aerobic combined with resistance training	8 3	0.53 0.75	[0.35, 0.71] [0.47, 1.04]	46.1% 69.0%	0.072 0.040	0.002**

*n*, Number of included studies; I^2^, Percentage of between-study heterogeneity; Pi, *p* value for within-subgroup heterogeneity test; *p*, *p* value for between-subgroup difference test. *p < 0.05, **p < 0.01.

**Table 6:** Effect of health status on PSPP total score and dimensions.

Dimension	Category	*n*	SMD	95% CI	*I^2^* (%)	Pi	*p*
Total score	Healthy individuals Sub-health population	6 7	1.12 0.66	[1.00, 1.23] [0.43, 0.89]	84.1% 37.2%	<0.001 0.159	0.001**
Physical self-worth	Healthy individuals Sub-health population	8 4	0.54 0.41	[0.45, 0.64] [0.13, 0.70]	62.4% 68.6%	0.010 0.023	0.404
Sport competence	Healthy individuals Sub-health population	8 4	0.64 0.81	[0.54, 0.73] [0.52, 1.11]	59.7% 33.0%	0.015 0.200	0.264
Physical condition	Healthy individuals Sub-health population	8 4	0.68 0.61	[0.56, 0.81] [0.25, 0.97]	35.1% 35.0%	0.148 0.202	0.708
Physical attractiveness	Healthy individuals Sub-health population	8 4	0.73 0.40	[0.64, 0.83] [0.12, 0.68]	74.2% 0.00%	<0.001 0.567	0.030*
Physical fitness	Healthy individuals Sub-health population	8 4	0.62 0.62	[0.53, 0.72] [0.33, 0.91]	74.7% 54.8%	<0.001 0.084	0.510

*n*, Number of included studies; I^2^, Percentage of between-study heterogeneity; Pi, *p* value for within-subgroup heterogeneity test; *p*, *p* value for between-subgroup difference test. *p < 0.05, **p < 0.01.

#### 3.6.1 Gender

The subgroup analyses presented in Table 4 suggest that gender may contribute to heterogeneity across several outcomes, including the total score, sport competence, physical condition, physical attractiveness, and physical fitness. In terms of pooled effect sizes, males appeared to derive greater benefits than females across most outcomes, including the total score (SMD = 1.15 vs. 0.55), sport competence (SMD = 0.82 vs. 0.48), physical condition (SMD = 0.83 vs. 0.48), physical attractiveness (SMD = 0.92 vs. 0.46), and physical fitness (SMD = 0.73 vs. 0.40). For physical self-worth, the pooled effect size was also higher in males (SMD = 0.65) than in females (SMD = 0.41), although the between-group difference did not reach statistical significance (*p* = 0.053).

This subgroup analysis was based on only three studies reporting male-only or disaggregated mixed-gender data and three studies reporting female-only data. Such a limited evidence base substantially reduces statistical power and increases the risk of spurious findings. Therefore, the observed gender differences should not be interpreted as definitive, but rather as preliminary signals that require confirmation in larger, gender-balanced trials.

#### 3.6.2 Exercise modality

The subgroup analyses presented in Table 5 suggest that intervention modality may influence the effect of exercise on physical self-esteem. In terms of pooled effect sizes, combined aerobic and resistance training appeared to be associated with larger effects than aerobic exercise alone across all examined outcomes, including the total score (SMD = 1.20 vs. 0.88), physical self-worth (SMD = 0.59 vs. 0.48), sport competence (SMD = 0.69 vs. 0.64), physical condition (SMD = 0.70 vs. 0.63), physical attractiveness (SMD = 0.89 vs. 0.53), and physical fitness (SMD = 0.75 vs. 0.43).

It should be emphasized that this subgroup analysis compared only two intervention modalities: combined aerobic–resistance training and aerobic exercise alone. Resistance training alone was not included as an independent subgroup because only one study evaluated this modality, which precluded reliable effect size pooling and formal comparison. Moreover, even between the two analyzable subgroups, the distribution of studies was markedly unbalanced, with three studies in the combined training subgroup and nine in the aerobic-only subgroup. Most importantly, no head-to-head randomized controlled trial directly comparing combined aerobic–resistance training versus aerobic exercise alone was identified in this review. All comparisons between the two modalities are therefore based on indirect between-study comparisons. Accordingly, the apparent advantage of combined training should not be interpreted as definitive evidence, but rather as a preliminary hypothesis that requires confirmation in large, well-powered head-to-head randomized controlled trials.

#### 3.6.3 Health status

The subgroup analyses presented in Table 6 suggest that health status may contribute to heterogeneity in the total score and the physical attractiveness dimension. In terms of pooled effect sizes, healthy participants appeared to derive greater benefits from exercise interventions than sub-healthy participants for the total score (SMD = 1.12 vs. 0.66) and physical attractiveness (SMD = 0.73 vs. 0.40). No significant between-group differences were observed for physical self-worth, sport competence, physical condition, or physical fitness (all *p* > 0.05).

It should be noted that the sub-healthy subgroup included only four studies and encompassed heterogeneous conditions, including obesity, depression, and physical frailty. This limited and clinically diverse evidence base, together with the indirect between-study nature of the comparison, means that the observed differences between healthy and sub-healthy participants should be interpreted as exploratory. Future research should conduct stratified analyses according to specific health conditions in order to generate more precise effect estimates.

### 3.7 Sensitivity analysis and publication Bias testing

Sensitivity analyses were performed using a leave-one-out approach. Sequential exclusion of each individual study did not materially alter the pooled effect sizes, indicating that the meta-analytic findings were generally robust and were not unduly driven by any single study.

**Figure 4:**
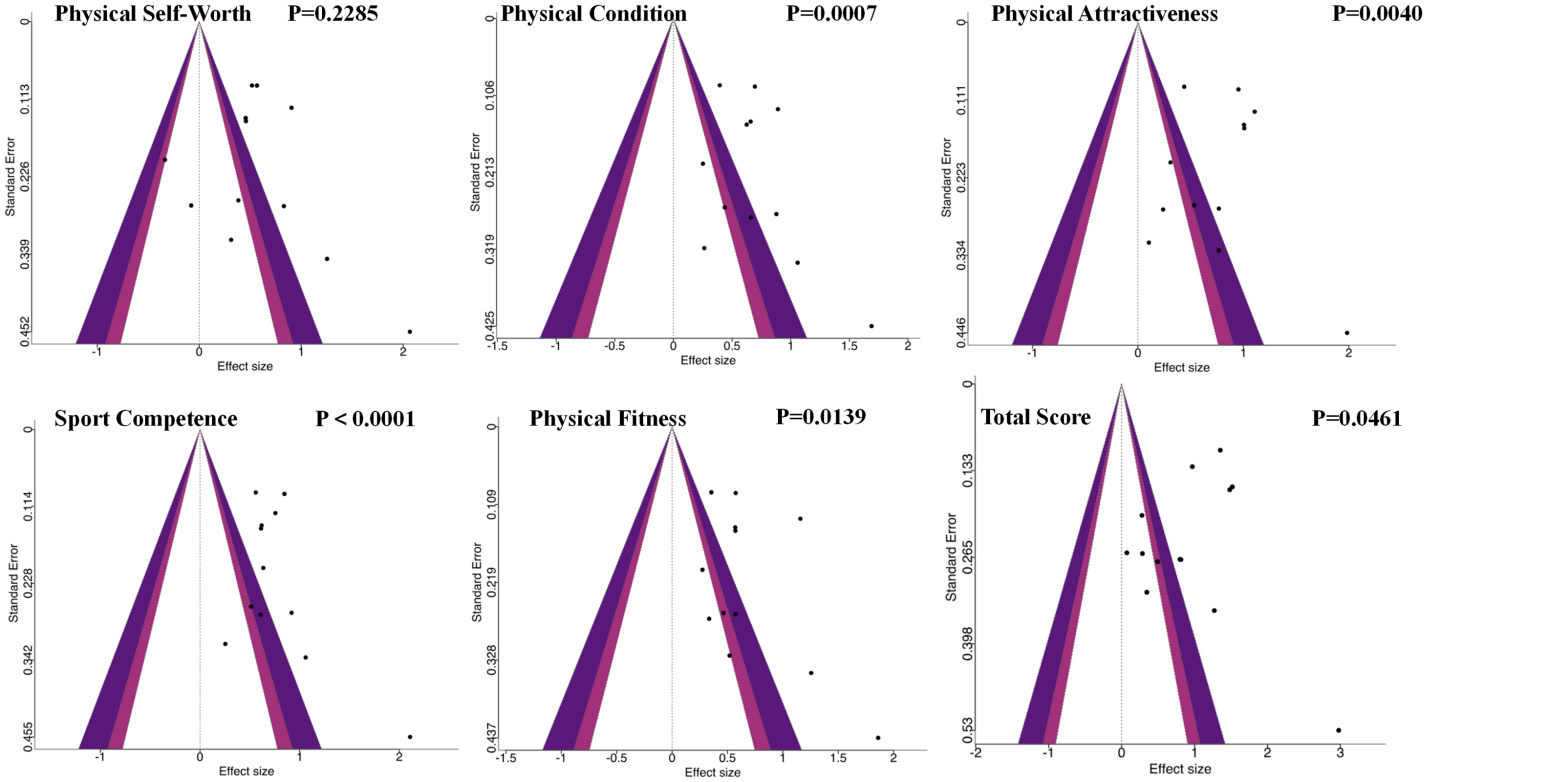
Funnel plot of publication bias test.

Publication bias and small-study effects were assessed through visual inspection of funnel plots and Egger’s linear regression test. Egger’s test indicated statistically significant asymmetry for the primary outcome of total physical self-esteem (*p* < 0.05), as well as for the secondary outcomes of sport competence (*p* < 0.001), physical condition (*p* = 0.0007), physical attractiveness (*p* = 0.0040), and physical fitness (*p* = 0.0139). No statistically significant evidence of publication bias was detected for physical self-worth (*p* = 0.2285) (Figure 4).

Duval and Tweedie’s trim-and-fill method was applied to outcomes with significant Egger’s test results to estimate the potential influence of publication bias. For the primary outcome of total physical self-esteem, six potentially missing studies were imputed, yielding an adjusted pooled effect size of SMD = 0.82 (95% CI [0.61, 1.03]). Although this corrected estimate was smaller than the original pooled effect (SMD = 0.95), it remained statistically significant (Z = 6.89, *p* < 0.001). Similar attenuation was observed for the secondary outcomes, although all adjusted estimates remained statistically significant. Specifically, the corrected SMD was 0.59 (95% CI [0.46, 0.72], *p* < 0.001) for sport competence, 0.58 (95% CI [0.47, 0.69], *p* < 0.001) for physical condition, 0.56 (95% CI [0.39, 0.73], *p* < 0.001) for physical attractiveness, and 0.54 (95% CI [0.38, 0.70], *p* < 0.001) for physical fitness.

Taken together, these findings indicate the presence of publication bias and small-study effects for most core outcomes in this meta-analysis, suggesting that the original pooled effect sizes may have been modestly overestimated. Nevertheless, all bias-adjusted effect sizes remained statistically significant, and the corrected estimates showed substantial overlap with the original confidence intervals. These results suggest that publication bias is unlikely to overturn the overall qualitative conclusion that exercise interventions are associated with improved physical self-esteem in college students, although the magnitude of the effect should be interpreted with caution.

### 3.8 Results of GRADE evidence quality assessment

The certainty of evidence for the six main outcomes was evaluated using the Grading of Recommendations Assessment, Development and Evaluation (GRADE) framework (Table 7).

**Table 7:** GRADE evidence quality assessment.

Outcome indicators	Number of studies	Sample size/*n*	Limitations	Inconsistency	Indirectness	Imprecision	Publication bias	Level of evidence
Total score	12	1,629	Downgraded by 1 level	Downgraded by 1 level	No downgrade	No downgrade	Downgraded by 1 level	Low
Physical self-worth	12	1998	Downgraded by 1 level	No downgrade	No downgrade	No downgrade	No downgrade	Moderate
Sport competence	12	1998	Downgraded by 1 level	No downgrade	No downgrade	No downgrade	Downgraded by 1 level	Low
Physical condition	12	1998	Downgraded by 1 level	Downgraded by 1 level	No downgrade	No downgrade	Downgraded by 1 level	Low
Physical attractiveness	12	1998	Downgraded by 1 level	No downgrade	No downgrade	No downgrade	Downgraded by 1 level	Low
Physical fitness	12	1998	Downgraded by 1 level	No downgrade	No downgrade	No downgrade	Downgraded by 1 level	Low

For the primary outcome of overall physical self-esteem (total score), the certainty of evidence was downgraded because of study limitations, substantial between-study inconsistency, and publication bias related to small-study effects. For the subdimensions of physical self-esteem, the certainty of evidence was downgraded by one level for study limitations, with additional downgrades for publication bias and/or between-study inconsistency applied where appropriate. Overall, the certainty of evidence ranged from low to moderate.

Taken together, the available evidence suggests that exercise interventions may improve physical self-esteem among college students. However, confidence in these findings is limited by methodological weaknesses in the included studies, between-study heterogeneity, and potential publication bias.

## 4 Discussion and analysis

This systematic review and meta-analysis synthesized evidence from 12 randomized controlled trials to evaluate the effects of exercise interventions on physical self-esteem among Chinese college students. The primary finding was that exercise interventions were associated with a statistically significant, large beneficial effect on overall physical self-esteem, along with moderate beneficial effects across all five subdimensions. However, the pooled effect estimate should be interpreted with caution because of the very high degree of between-study heterogeneity (*I^2^* = 79%). Subgroup analyses further suggested that gender, exercise modality, and health status may moderate intervention effects, although these findings remain preliminary and should be regarded as hypothesis-generating rather than conclusive.

### 4.1 Analysis of the impact of exercise on physical self-esteem

Our pooled analysis showed that exercise interventions were associated with significant improvements in overall physical self-esteem among college students (SMD = 0.95), as well as in all examined subdimensions (SMD = 0.55–0.68). It should be emphasized that none of the mechanistic pathways discussed below were directly assessed in the studies included in this meta-analysis. Accordingly, the following interpretations should be regarded as theoretical explanations for the observed associations rather than as confirmed causal mechanisms.

Several plausible pathways may underlie these beneficial effects, broadly encompassing physiological, psychological, and social processes. From a physiological perspective, exercise may enhance physical self-esteem by improving physical fitness, optimizing body composition, and promoting general health. Previous studies have shown that regular aerobic exercise, resistance training, and combined training can reduce appetite and serum leptin levels, increase lean body mass, and improve body composition (Mann et al., 2014; Migueles et al., 2023; Smart et al., 2025). Exercise has also been linked to broader improvements in metabolic efficiency, energy regulation, and physical functioning (Febbraio, 2017; O’Gorman et al., 2020; Viru and Smirnova, 1995). Together, these changes may create favorable objective conditions for more positive evaluations of the physical self.

From a psychological perspective, the positive effects of exercise on physical self-esteem may be related to enhanced self-efficacy, correction of maladaptive body-related cognitions, and alleviation of negative affect. Self-efficacy refers to individuals’ beliefs in their capacity to successfully perform specific behaviors and is closely related to confidence and self-esteem (Bandura, 1993). A meta-analysis of 36 randomized controlled trials found that exercise interventions improved perceived self-efficacy, with a moderate-to-large effect size (Hedges’ g = 0.68), across a range of populations, including adults with obesity and chronic disease (Baghbani et al., 2023). In addition, low physical self-esteem may partly reflect distorted perceptions of the body rather than objective physical deficits. Previous evidence suggests that even low-intensity exercise can improve overall body image perception across diverse populations (Singh et al., 2025). Such cognitive changes may help reduce self-evaluative biases related to the body. Exercise may also benefit physical self-esteem by reducing negative emotions such as anxiety and depression. Negative emotional states can heighten attention to perceived bodily flaws and thereby undermine body confidence (Kocur and Jach, 2024). By contrast, exercise has been associated with improved mood and positive affect, potentially through neurobiological pathways involving serotonin, endorphins, and dopamine, which may in turn promote more favorable perceptions of one’s physique (Meeusen and De Meirleir, 1995).

Social processes may also contribute to the observed benefits. According to social identity theory, self-evaluations are shaped not only by personal perceptions but also by feedback and norms within the surrounding social environment. Positive social signals conveyed through peer interaction may reinforce favorable perceptions of sport competence and physical condition, thereby compensating for the limitations of solitary self-appraisal. Previous studies have shown that social support, a sense of group belonging, and supportive peer behaviors in sport settings are positively associated with exercise motivation and self-esteem, particularly in team-based activities (Eime et al., 2013).

Taken together, these physiological, psychological, and social pathways may help explain why exercise interventions were associated with improved physical self-esteem in the present review. Nevertheless, none of these mechanisms were directly measured in the included studies, and they should therefore be interpreted as plausible hypotheses rather than confirmed explanations.

### 4.2 Analysis of gender effects on intervention outcomes

Our subgroup analyses suggested that males showed larger pooled effects than females for overall physical self-esteem (SMD = 1.15 vs. 0.55), sport competence (SMD = 0.82 vs. 0.48), physical fitness (SMD = 0.73 vs. 0.40), physical attractiveness (SMD = 0.92 vs. 0.46), and physical condition (SMD = 0.83 vs. 0.48). However, these findings should be interpreted with caution. They reflect exploratory between-study comparisons rather than evidence of inherent or essential sex differences. As noted in the Results section, the available subgroup evidence is limited by substantial methodological constraints and does not support causal inference. The observed differences may instead reflect variation in training responsiveness, prior exercise experience, intervention content, and sociocultural context.

One possible explanation is that female participants may have entered the interventions with lower baseline physical self-esteem than male participants. Previous research has shown that female high school and college students report higher levels of social physique anxiety and lower physical self-esteem than males (Hagger and Stevenson, 2010; Manzano-Sánchez et al., 2022), which may have reduced the extent of detectable improvement within the relatively short intervention periods (8–16 weeks) used in most included studies. In addition, previous evidence suggests that males may experience larger absolute gains in muscle mass and fat reduction following structured exercise (Refalo et al., 2025; Noh et al., 2024), which could translate into more noticeable physical changes and, consequently, more immediate improvements in self-perception.

A second possible explanation relates to the nature of the interventions themselves. Several of the included studies focused on activities such as basketball and strength training, in which female participants may have had less prior experience or lower initial sport confidence (Zuk et al., 2021). This may have contributed to lower initial self-efficacy and reduced engagement with the intervention. Notably, none of the included studies implemented sex-tailored exercise programs, which may have limited intervention responsiveness among female participants.

Sociocultural influences may also have played a role. Compared with males, females are often exposed to stronger social pressure to conform to a narrow thin-ideal standard, which may reduce the extent to which functional or muscular improvements are translated into more positive physical self-evaluations (Carey et al., 2014). As a result, even beneficial exercise-induced physical adaptations may not necessarily produce equivalent gains in physical self-esteem. This interpretation, however, remains hypothetical, as these mechanisms were not directly examined in the included studies.

Finally, several methodological limitations should be acknowledged. Reporting of sex-stratified data was inconsistent, and few studies accounted for potentially important confounding variables such as prior exercise experience, baseline body image concerns, or sociocultural influences. Future research should therefore use larger, sex-balanced samples and more rigorously account for these factors when examining potential sex differences in intervention effects.

### 4.3 Analysis of the impact of different exercise modalities on intervention outcomes

Our subgroup analyses suggested that combined aerobic and resistance training may produce more favorable effects than aerobic exercise alone on overall physical self-esteem and its subdimensions. However, this finding should be interpreted with considerable caution. It is based on a small number of combined-training studies (*n* = 3), substantial imbalance in subgroup sizes, and indirect between-study comparisons, and therefore cannot be regarded as definitive evidence. The observed pattern may nevertheless reflect differences in the physiological and psychological effects of these exercise modalities.

From a physiological perspective, combined training may offer complementary benefits that are less likely to be achieved through aerobic exercise alone. For example, a study involving adolescents with overweight found that combined training did not produce greater improvements in maximal oxygen uptake (VO₂_max_) than aerobic training alone, but did confer clearer benefits in lower-body explosive power and core muscular endurance (Yugang et al., 2020). Although aerobic exercise is effective for reducing body fat and improving cardiorespiratory fitness, it may have limited effects on increasing muscle mass and muscle cross-sectional area (Ross et al., 2024). By contrast, combined aerobic and resistance training may simultaneously support fat loss, aerobic capacity, muscular development, and physical fitness. Such concurrent improvements in body composition and physical function may, in turn, promote more favorable perceptions of body shape and physical capability, thereby contributing to higher physical self-esteem.

Psychologically, combined training may also offer advantages over single-modality aerobic exercise. The greater variety of activities involved may increase enjoyment and engagement while reducing the monotony and fatigue that can accompany repetitive aerobic training. This may encourage sustained participation and reinforce positive self-perceptions over time. Nevertheless, only one included study evaluated resistance training alone. As a result, resistance-only interventions could not be meaningfully included in the comparative subgroup framework. The present analysis therefore allows comparison only between combined aerobic–resistance training and aerobic exercise alone, rather than among all three major training modalities. Further well-powered trials directly comparing aerobic, resistance, and combined training are needed to clarify their relative effects on physical self-esteem.

### 4.4 Analysis of the impact of health status on intervention outcomes

Our subgroup analyses suggested that participants’ health status may influence intervention effects. Specifically, exercise interventions appeared to yield larger benefits for overall physical self-esteem and physical attractiveness in healthy participants than in sub-healthy participants. However, this finding should be interpreted cautiously. From a methodological perspective, the sub-healthy subgroup included only four studies and encompassed heterogeneous conditions such as obesity, depression, and physical frailty. Given the small number of studies and the heterogeneity of the included conditions, this subgroup differences should be regarded as exploratory. No significant between-group differences were observed for physical self-worth, sport competence, physical condition, or physical fitness. The observed pattern may nevertheless reflect differences in physiological responsiveness and psychological readiness to benefit from exercise.

One possible explanation is that healthy participants may experience more rapid and coordinated improvements in body composition and physical fitness, which could more readily translate into enhanced physical attractiveness and overall physical self-esteem. In addition, they may be less burdened by longstanding negative body perceptions and therefore more likely to interpret modest exercise-related gains in a positive way. Successful completion of exercise tasks may further reinforce self-efficacy, thereby amplifying intervention effects.

By contrast, sub-healthy participants may show slower or less perceptible changes over a short intervention period. For example, participants with obesity may experience limited short-term changes in body shape, those with physical frailty may progress more slowly in physical capacity, and those with depression may have greater difficulty perceiving or internalizing positive bodily changes. Given that most included interventions lasted only 8–16 weeks, the full benefits of exercise may not have been fully captured in these populations. In addition, pre-existing physical or psychological limitations may increase the likelihood of exercise-related setbacks, such as difficulty completing prescribed movements or early fatigue, which could undermine self-efficacy and reduce intervention responsiveness.

These findings should also be viewed in light of several methodological limitations. The included studies did not apply a uniform definition of “sub-health,” and substantial variation existed across studies with respect to condition severity, duration, and baseline activity level. Accordingly, the interpretations offered here remain theoretical and were not directly tested in the included studies. Future research should include larger samples of sub-healthy participants, conduct stratified analyses according to specific health conditions, and adopt longer intervention periods to generate more precise estimates of effect.

### 4.5 Research limitations

This study has several limitations. First, substantial between-study heterogeneity was observed for the primary outcome (*I^2^* = 79%), which reduces the precision of the overall pooled effect estimate. Although we identified several potential moderators—namely gender, exercise modality, and health status—that accounted for a substantial proportion of this heterogeneity, some residual variance remained unexplained, partly because of limited reporting of study-level characteristics in the included studies.

Second, the coverage of exercise modalities and intensities was incomplete. All included studies focused on moderate-intensity exercise, and no study examined low-intensity or high-intensity interval exercise, limiting our ability to evaluate dose–response relationships between exercise intensity and physical self-esteem. In addition, only one study investigated resistance training alone, which precluded a comprehensive comparison among aerobic training, resistance training, and combined training.

Third, the statistical power of the subgroup analyses was limited. The relatively small number of included studies reduced the power of analyses stratified by gender, health status, and exercise modality and may have increased the risk of false-negative findings in some moderator analyses. Accordingly, all subgroup findings should be interpreted as exploratory and hypothesis-generating.

Fourth, publication bias was detected for most core outcomes. Although the overall qualitative conclusion remained unchanged after trim-and-fill adjustment, the exclusion of grey literature may have contributed to a modest overestimation of the intervention effect size.

Finally, the generalizability of the findings may be limited. All included studies were conducted in China, and the participants were restricted to college students, primarily aged 18–24 years. Therefore, the findings may not be directly generalizable to other age groups, educational settings, or cultural contexts.

## 5 Conclusion

Current evidence suggests that exercise interventions may improve overall physical self-esteem and its subdimensions among Chinese college students. Exploratory subgroup analyses further suggest that larger benefits may be observed in males, in participants receiving combined aerobic–resistance training, and in healthy individuals. However, these subgroup findings remain preliminary and hypothesis-generating, as they are based on a limited number of studies, imbalanced subgroup sizes, and indirect between-study comparisons. Confirmation in larger, well-powered randomized controlled trials is therefore needed.

Overall, exercise appears to be a promising strategy for enhancing physical self-esteem among college students and may also confer broader mental health benefits. Future research should adopt longitudinal designs and incorporate potential mediating variables to clarify the mechanisms underlying these effects.

## Data Availability

The data analyzed in this systematic review and meta-analysis are derived from previously published peer-reviewed studies. All included studies/data are cited in the reference list of this article. Further inquiries can be directed to the corresponding author(s).
